# A Large Anisotropic Enhancement of the Charge Carrier Mobility of Flexible Organic Transistors with Strain: A Hall Effect and Raman Study

**DOI:** 10.1002/advs.201901824

**Published:** 2019-11-13

**Authors:** Hyun Ho Choi, Hee Taek Yi, Junto Tsurumi, Jae Joon Kim, Alejandro L. Briseno, Shun Watanabe, Jun Takeya, Kilwon Cho, Vitaly Podzorov

**Affiliations:** ^1^ Department of Physics Rutgers University Piscataway NJ 08854 USA; ^2^ School of Materials Science and Engineering and Engineering Research Institute Gyeongsang National University Jinju 52828 Korea; ^3^ International Center of Materials Nanoarchitectonics National Institute for Materials Science (NIMS) 1‐1 Namiki Tsukuba 305‐0044 Japan; ^4^ Material Innovation Research Center (MIRC) and Department of Advanced Materials Science Graduate School of Frontier Sciences The University of Tokyo 5‐1‐5 Kashiwanoha Kashiwa Chiba 277‐8561 Japan; ^5^ National Institute of Advanced Industrial Science and Technology (AIST) The University of Tokyo Advanced Operando‐Measurement Technology Open Innovation Laboratory (OPERANDO‐OIL) AIST, 5‐1‐5 Kashiwanoha Kashiwa Chiba 277‐8561 Japan; ^6^ Department of Polymer Science and Engineering University of Massachusetts Amherst Amherst MA 01003 USA; ^7^ Center for Advanced Soft Electronics and Department of Chemical Engineering Pohang University of Science and Technology (POSTECH) Pohang 37673 Republic of Korea; ^8^ Present address: Department of Chemistry The Pennsylvania State University University Park PA 16803 USA

**Keywords:** flexible electronics, mobility, organic transistors, Raman, strain

## Abstract

Utilizing the intrinsic mobility–strain relationship in semiconductors is critical for enabling strain engineering applications in high‐performance flexible electronics. Here, measurements of Hall effect and Raman spectra of an organic semiconductor as a function of uniaxial mechanical strain are reported. This study reveals a very strong, anisotropic, and reversible modulation of the intrinsic (trap‐free) charge carrier mobility of single‐crystal rubrene transistors with strain, showing that the effective mobility of organic circuits can be enhanced by up to 100% with only 1% of compressive strain. Consistently, Raman spectroscopy reveals a systematic shift of the low‐frequency Raman modes of rubrene to higher (lower) frequencies with compressive (tensile) strain, which is indicative of a reduction (enhancement) of thermal molecular disorder in the crystal with strain. This study lays the foundation of the strain engineering in organic electronics and advances the knowledge of the relationship between the carrier mobility, low‐frequency vibrational modes, strain, and molecular disorder in organic semiconductors.

## Graphical abstract

This paper reports Hall effect and Raman measurements of organic semiconductors as a function of uniaxial mechanical strain. It reveals a strong, anisotropic modulation of the intrinsic charge carrier mobility of single‐crystal rubrene transistors with strain, showing that the performance of organic circuits can be enhanced by up to 100% with only 1% of compessive strain.

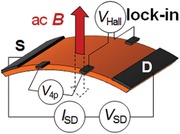

## 1 Introduction

Organic semiconductors are considered for numerous applications in flexible, wearable and disposable electronics due to their interesting optoelectronic properties, versatile processability and favorable mechanical properties.^1^, ^2^, ^3^, ^4^ In order to realize this potential, one must have an understanding of the effects of applied strain on the intrinsic charge carrier mobility, μ, of these materials in flexible devices. While engineering of bendable organic field‐effect transistors (OFETs) based on amorphous or polycrystalline materials has advanced, little effort has been made toward our understanding of the intrinsic effects of applied strain on the trap‐free charge carrier mobility. By “intrinsic” we imply the reversible modulation of the trap‐free charge carrier mobility of organic semiconductors with a strain‐induced elastic deformation of the crystal lattice. Studies of OFETs' stability on bending, while being important for applications,^3^, ^5^, ^6^ are not our focus here. Understanding the intrinsic mobility–strain relationship is not only important for high‐performance flexible organic electronics, but also necessary for implementing strain engineering in future applications, including motion and tactile sensors and actuators.

Charge transport in “soft” van der Waals molecular solids is governed by the three key factors. The first factor is the equilibrium position of molecules in a crystal lattice that defines the average transfer integrals between the adjacent molecules. Large transfer integrals promote charge carrier delocalization and formation of electronic bands, thus leading to higher carrier mobilities.^7^ The second factor is the thermal molecular motion responsible for the off‐diagonal thermal disorder in transfer integrals.^8^, ^9^ In molecular crystals, this intrinsic disorder may lead to a breakdown or weakening of the extended band and occurrence of localized states near the band edges (the tail states), thus causing transient carrier localization.^10^ It has been recently suggested that molecular vibrations most significantly limiting the charge carrier transport and reducing *µ* are among the low‐frequency (1 – 300 cm^−1^) modes in Raman spectra of organic semiconductors (see, e.g., ref. 11 and references therein). However, a direct link between the charge carrier mobility and these Raman modes has not been experimentally established. In rubrene, these modes were theoretically shown to be the result of a combination of inter‐ and intramolecular vibrations.^12^ Finally, formation of small polarons (the carriers “dressed” in a polarization cloud) can also lead to a breakdown of the band and a reduced charge mobility.^13^ In small‐molecule organic semiconductors, these competing factors may depend on molecular structure, molecular packing, as well as external conditions (temperature, pressure, strain). As a consequence, we have systems with different transport properties: some materials exhibit a nearly perfect band‐like transport with high mobility, such as, for example, rubrene (5,6,11,12‐tetraphenyltetracene, C_42_H_28_),^14^ as well as alkyl end‐capped benzothieno‐benzothiophene and dinaphto‐thienothiophene (C_8_‐BTBT and C_10_‐DNTT),^15^, ^16^ some are characterized with a purely hopping conduction and low *µ*, and others show a “mixed” transport, in which band‐like and hopping states coexist, as has been recently shown via high‐resolution *ac*‐Hall effect measurements of OFETs.^17^

Both π–π interactions and thermal disorder can be delicately affected by intermolecular distances in crystals. For instance, in rubrene, the equilibrium transfer integral is a steep function of the relative displacement of the molecules,^7^ and it was experimentally shown that application of a high hydrostatic pressure results in a significant increase of the carrier mobility of single‐crystal rubrene OFETs due to a reduction of the intermolecular distances.^18^, ^19^ It was also hypothesized that hydrostatic pressure or strain can reduce off‐diagonal disorder by suppressing thermal fluctuations.^20^

The most convenient experimental approach to study these intrinsic effects in organic semiconductors is to apply uniaxial mechanical strain to flexible single‐crystal transistors. The advantages of strain‐variable measurements over the experiments under a hydrostatic pressure are as follows: a) they are technically much easier to perform (a high‐pressure anvil cell is not required), b) one can apply either compressive or tensile strains, c) strain can be applied along different crystallographic directions within the basal plane of the device, and d) there is practically no limitation on device size. Reyes‐Martinez et al. recently demonstrated that wrinkling instability could be used as a tool to modulate the conventional two‐probe mobility (the so‐called longitudinal mobility), *µ*_FET_, in rubrene single‐crystal OFETs with strain.^21^, ^22^ Strain induced modulations of *µ*_FET_ were also recently demonstrated in solution‐processed crystalline 3,11‐didecyldinaphto[2,3‐d:2′,3′‐d′]benzo[1,2‐b:4,5‐b′]dithiophene (C_10_‐DNBDT‐NW) OFETs.^20^ While such strain variable measurements in single‐crystal OFETs have started to emerge,^20^, ^21^, ^22^, ^23^, ^24^ to our knowledge there are no studies of the Hall effect versus strain in organic semiconductors. Such studies would reveal the response of the intrinsic (trap free) carrier mobility to strain, because Hall effect in highly ordered crystalline organic semiconductors tends to be sensitive primarily to the mobile carriers moving in extended states.^15^, ^17^, ^25^, ^26^

We emphasize that while applied studies show that polycrystalline or amorphous OFETs can withstand a considerable bending without mobility degradation,^3^, ^6^ there are only a handful of studies of the impact of strain on the charge transport in single‐crystal organic semiconductors.^20^, ^21^, ^22^, ^23^, ^24^ Given the relevance of high‐mobility crystalline OFETs for organic electronics,^2^, ^4^, ^20^, ^27^, ^28^ addressing the intrinsic effect of strain on *µ* due to the elastic lattice deformation is very important. Rubrene, in particular, stands out as a relevant test system because of its well‐defined surface morphology,^29^ crystal structure,^30^ interesting optoelectronic properties,^4^ ideal field‐effect transistor behavior,^14^, ^31^ as well as a band‐like charge transport in extended states with a high room‐temperature mobility.^25^, ^26^ In addition, it has been recently shown that extremely thin (0.1–1 µm) and flexible rubrene crystals of macroscopic lateral dimensions (up to 1 cm) can be grown by physical vapor transport and integrated into OFETs.^21^, ^22^

Here, we quantify the strain effect on mobility by a strain factor, *g*, which is a relative change of the carrier mobility, Δ*µ*/*µ*_0_, per unit of the applied strain ε, occurring in a FET on bending: *g* ≡ |δ(Δ*µ*/*µ*_0_)/*δε*|. Here, Δ*µ*(ε) = *µ*(ε) − *µ*_0_ is the difference between the mobility *µ*(ε) of a device under strain ε and the mobility *µ*_0_ of the unstrained device. Both Δ*µ*/*µ*_0_ and ε in this definition are expressed either in percent or as decimal fractions. For instance, a strain factor of 100 would mean that the device under a compressive strain of 1% has a carrier mobility 100% greater than that of the unstrained device. Recent measurements of strain factors in crystalline OFETs have led to significant ambiguity in results. For instance, Wang et al. and Matta et al. observed a relatively weak modulation of *µ* with strain in two‐probe transistor measurements, yielding *g* = 20 and 6, respectively.^23^, ^24^ In contrast, Reyes‐Martinez et al. reported a much stronger enhancement of *µ* with compressive strain in measurements of “wrinkled” two‐probe FETs, with *g* up to 250.^22^ Besides rubrene, the mobility–strain relationship was also evaluated in solution‐processed single‐crystalline C_8_‐DNBDT‐NW OFETs, also via longitudinal FET transport measurements, in which *g* ≈ 20 was reported.^20^ These widely varying results on the apparent strain‐induced mobility variations could be attributed to potential drawbacks of two‐probe FET measurements, related for instance to the modulations of the metal contact work function with strain, recently observed by Wu et al.^32^ In wrinkled devices, extraction of the net strain is not straightforward and has to rely on complex modeling. Finally, the conventional (longitudinal) FET transport measurements cannot easily distinguish between the intrinsic (not dominated by trapping) and extrinsic (dominated by trapping) conduction regimes.^25^

To address these issues, here we employ the Hall effect studies coupled with four‐probe FET measurements of ultrathin single‐crystal organic transistors subjected to a calibrated uniaxial strain. These flexible four‐probe FETs allow us to avoid the artifacts caused by disorder and reveal the intrinsic mobility–strain relationship (for details on four‐probe OFETs, see, e.g., ref. 14 and references therein). The Hall effect measurements were carried out via the high‐resolution *ac*‐Hall methodology capable of reliably detecting Hall signal and assessing the intrinsic (Hall) carrier mobilities in OFETs with *µ* well below 1 cm^2^ V^−1^ s^−1^.^33^

Overall, we found that both the longitudinal FET mobility, *µ*_FET_, and the Hall mobility, *µ*_Hall_, of rubrene single‐crystal OFETs exhibit a strong anisotropic dependence on strain, ε, with the anisotropy in *µ*(ε) response consistent with the molecular packing of the crystal. For charge transport along the high‐mobility axis of rubrene (here, the ***b***‐axis), we measured the *intrinsic* strain factor of *g* = 70–110 for the strain applied along the same transport direction, and *g* ≈ 0 for the strain applied along the orthogonal low‐mobility direction (here, the ***a***‐axis) of the crystal. These values are among the highest strain‐induced modulations of *µ* (and the greatest strain effect anisotropy) reported for organic semiconductors.

## 2 Results

### 2.1 Flexible Single‐Crystal OFETs and Measurement Approaches

**Figure 1:**
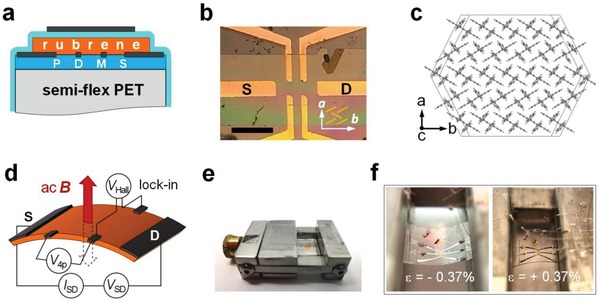
Flexible single‐crystal organic transistors for FET and Hall measurements under strain. a) A cross‐sectional device structure: a 40 µm thick semirigid PET substrate coated with 1 µm thick PDMS adhesion layer supports evaporated Ti/Au contacts and an ultrathin rubrene single crystal; a parylene‐*N* gate insulator and an Ag gate are deposited on top of the structure. b) An optical microphotograph of a device consisting of a 260 nm thick rubrene single crystal laminated on a PET/PDMS substrate with four‐probe contacts (scale bar is 0.3 mm). The crystal is oriented with its high‐mobility ***b***‐axis along the source–drain transport direction (the molecular packing and the crystal axes are schematically shown). The strain can be applied either along ***b***‐ or ***a***‐axes. c) A sketch of molecular packing on the used here largest natural facet (the (***a***,***b***)‐facet) of orthorhombic rubrene. d) A sketch of *ac*‐Hall effect measurements of a flexible organic single crystal under strain. An *ac* magnetic field and detection of Hall voltage with a lock‐in amplifier are used to achieve high signal/noise ratio (for details on *ac*‐Hall effect technique, see the Experimental Section and ref. 33). e) A photograph of the custom‐designed miniature strain stage with a micrometric screw for calibrated strain application (the height of the stage is 5 mm) with a device loaded in it. f) Photographs of a device under compressive (left) and tensile (right) strains.

The experimental arrangement is shown in **Figure** **1**. The devices consist of ultrathin flexible rubrene single crystals laminated on semirigid 40 µm thick polyethylene terephthalate (PET) substrates precoated with 1 µm thick polydimethylsiloxane (PDMS) layer for better crystal adhesion. Before laminating a crystal, Ti/Au contacts in four‐probe geometry are sputtered on PDMS surface via a contact shadow mask. A parylene‐*N* gate insulator and Ag gate are deposited on top of the structures (Figure 1a,b). The (orthorhombic) rubrene crystals are aligned to the contacts, so that their high‐mobility ***b***‐axis is the transport direction (Figure 1b,c). The crystal axes are identified via a polarized optical transmission microscopy (Section S1, Supporting Information). For Hall measurements, an *ac* magnetic field of an r.m.s. magnitude *B* = 0.23 T is applied perpendicular to the crystal's (***a***,***b***)‐facet, when the FET is on, and *ac*‐Hall voltage is measured by a lock‐in amplifier (Figure 1d).^33^ Uniaxial strain parallel (or perpendicular) to the charge transport direction can be applied by bending the semirigid PET substrate supporting the device in a custom‐designed miniature strain stage (Figure 1e). The substrate can be bent inward (ε < 0, compressive strain) or outward (ε > 0, tensile strain) from its initial planar geometry (ε = 0, unstrained), as shown in Figure 1f. The strain magnitude is defined as $\varepsilon \equiv \frac{t}{2R}\times \;100\%$, where *t* is the net thickness of the substrate (*t* = 41 µm), and *R* is the bending radius (Section S2, Supporting Information).^34^ The Hall mobility, *µ*_Hall_, is obtained from the Hall voltage, *V*_Hall_, and the longitudinal four‐probe voltage, *V*_4p_, simultaneously measured in a device carrying a source–drain current, *I*_SD_, in the accumulation channel, while the device is in the magnetic field *B*, according to

(1) $$\mu_{\mathrm{Hall}}\equiv \frac{D}{W}\cdot \frac{1}{B}\cdot \frac{V_{\mathrm{Hall}}}{V_{4p}}$$

where *D* is the distance between the voltage probes (in the longitudinal direction) in the four‐probe contact geometry and *W* is the channel width (roughly the same as the distance between the Hall voltage probes). The strain factor *g* is determined from the experimental *µ*(ε) dependence as

(2) $$g\equiv \frac{1}{\mu_{0}}\cdot \frac{d\mu }{d\varepsilon }\times \;100\%$$

where subscript “0” refers to the value for the unstrained (ε = 0) device, and the strain ε is in %.

### 2.2 Measurements of FET and Hall Mobility under Strain

**Figure 2:**
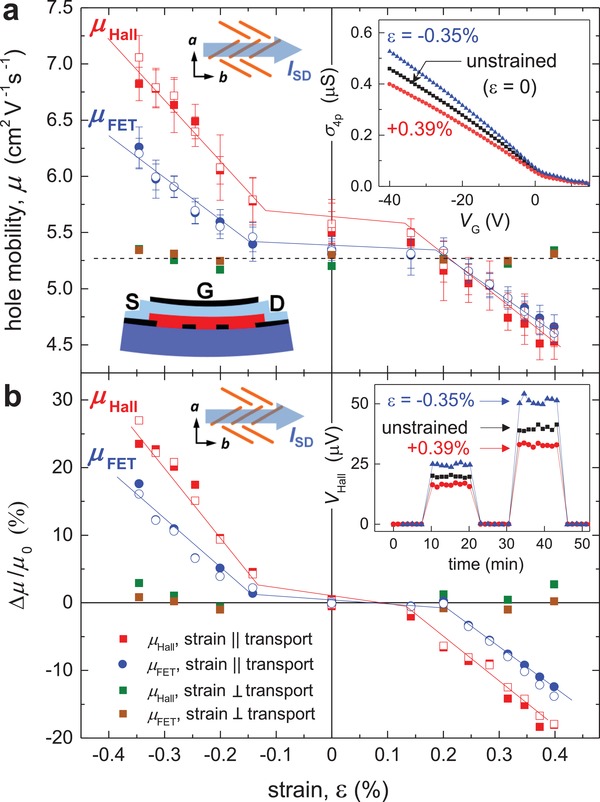
FET and Hall measurements of a single‐crystal rubrene OFET as a function of uniaxial strain. a) The absolute FET and Hall mobilities (blue circles and red squares, respectively). The insets schematically show the molecular packing and the transport direction, the gated four‐probe OFET structure (dark blue: bendable substrate, red: rubrene single crystal, light blue: parylene‐*N* gate dielectric, black: electrodes), as well as the examples of the transconductance characteristics of a strained and unstrained OFET in the linear regime (*V*_SD_ = 2 V). b) The corresponding relative change in the FET and Hall mobilities. The inset shows the examples of the Hall voltage recorded in a strained and unstrained OFET (shown for the two sequentially applied source–drain excitation currents, *I*_SD_ = 130 and 260 nA). In both panels, the charge transport direction is along the high‐mobility ***b***‐axis of the crystal, while strain is applied parallel (red and blue symbols) or perpendicular (green and brown symbols) to this transport direction. Solid and open symbols correspond to the measurements taken on increasing or decreasing strain magnitude, respectively. Negative or positive ε corresponds to a compressive or tensile strain, respectively. *µ*_FET_ is obtained in the linear regime of FET operation from the extended linear portions of transconductance characteristics (at |*V*_G_| >> *V*_SD_ = 2 V). *µ*_Hall_ is measured at *V*_G_ = – 40 V. Device parameters: parylene‐*N* thickness is 1.9 µm (*C*_i_ = 1.235 nF cm^−2^), channel length *L* = 0.3 mm, channel width *W* = 0.1 mm, the center‐to‐center distance between the voltage probes is *D* = 0.1 mm, and the probe width is *t* = 48 µm. For details on the correction factors due to the longitudinal channel shunting effect, see ref. 46. These data reveal a large, anisotropic effect of strain on the ***b***‐axis mobility of rubrene, with the strain factor of *g* = 70–110 for the strain along the ***b***‐axis, and *g* ≈ 0 for the strain along the ***a***‐axis.

FET and Hall effect measurements in these flexible rubrene OFETs as a function of uniaxial strain are shown in **Figure** **2** (details are given in the caption). The absolute values of the longitudinal FET and Hall mobilities, *µ*_FET_ and *µ*_Hall_, are shown in Figure 2a, while the corresponding relative mobility change, Δ*µ*/*µ*_0_, is shown in Figure 2b. The insets in the top panel show a schematic device structure and examples of the four‐probe FET transfer characteristics, σ_4p_(*V*_G_), of one of these FETs in the linear regime (*V*_SD_ = 2 V) for an unstrained (ε = 0), compressed (ε = −0.35%) and stretched (ε = 0.39%) states of the device. It is worth noting that the device is functioning as a classic high‐performance OFET with a linear transconductance and a nearly zero onset voltage. The mobilities, *µ*_FET_ and *µ*_Hall_, are extracted from the extended linear portion of the curve at sufficiently high negative *V*_G_, where the mobility is gate voltage and carrier density independent. For more details on reliable extraction of carrier mobility in FETs, see ref. 14. It can be clearly seen from this inset that the transconductance slope changes with strain. The inset in the lower panel shows examples of as‐measured Hall voltage in this OFET for two levels of *I*_SD_ excitation and the same three values of strain, also clearly showing that *V*_Hall_ is sensitive to strain. In addition to the strain along the transport direction (along the molecular stacks), we have also evaluated the response of the FET and Hall mobilities to the strain applied perpendicular to the transport direction (perpendicular to the stacks).

Several important observations can be made from these *µ*_FET_(ε) and *µ*_Hall_(ε) measurements. Both Hall and FET measurements yield consistent results, with similar absolute values of *µ*, which is increasing (decreasing) with compressive (tensile) strain applied along the transport direction. The mobility–strain relationship *µ*(ε) shows a weak non‐linearity around ε = 0 that may look like a threshold. Currently, the origin of this plateau‐like feature at |ε| ≤ 0.15% is unclear, but it might be due to a partial stress relief in the PDMS elastomer used here for better crystal adhesion, or due to a very small amount of the initial strain being accommodated by residual structural defects of the crystal. Nevertheless, this should not affect our measurements of the strain factor that rely on the derivative, *δμ*/*δε*, of the quasi‐linear portion of *µ*(ε) curves. The maximum strain in our measurements (±0.40%) is dictated by higher noise in *V*_Hall_ occurring for ε outside of this range, which is perhaps due to the generation of additional defects under greater strains (for as‐measured *V*_Hall_ within this ε range see Section S3 in the Supporting Information). We have verified the elastic nature of the strain effect on mobility by first carrying out the measurements up to the maximum value of |ε| and then reducing |ε| step‐by‐step to several lower values, which yielded similar *µ*(ε) data (Figure 2), thus showing that within the error‐bar of these measurements the data are reproducible on strain reversal. Anisotropy of the strain effect, consistent with the molecular packing in rubrene, is observed. The strain factors for FET and Hall mobilities are *g*_FET_ ≈ 70 and *g*_Hall_ ≈ 110 for the strain applied along the transport direction (along the molecular stacks), that is, *µ* increases by nearly a factor of two at a compressive strain of only 1% applied along the high‐mobility ***b***‐axis. On the contrary, no changes in *µ* are observed for the strain applied perpendicular to the transport direction (perpendicular to the stacks, which is the low‐mobility ***a***‐axis). Overall, *µ*_Hall_ and *µ*_FET_ are close to each other, which is indicative of a nearly trap‐free (intrinsic) charge transport operation of these OFETs.

### 2.3 Eliminating Possible Artifacts

Spurious effects are rigorously minimized in our experiment. For example, inhomogeneous strain distribution due to strain accommodation by defects and/or metal contacts is unlikely, because a) very thin (100–300 nm thick) and uniform single crystals are used, b) strain is governed by a well‐defined macroscopic bending radius of a much thicker (41 µm thick) substrate. The use of monolithic single crystals eliminates possible effects of grain boundaries. A good match between the four‐probe FET and Hall measurements, as well as the reversibility of the observed mobility–strain dependence, indicate that our devices are neither contact‐limited nor trap‐dominated, and we mostly probe the intrinsic (trap‐free) charge transport properties of the crystals. In addition, within the strain range used here (up to ±0.4%), no observable delamination, wrinkling or cracking were detected in our devices. Since Hall effect directly measures the mobile carrier density, without relying on certain gate‐channel capacitance, possible modulation of *C*_i_ with strain does not affect our measurements. Flexo‐ or piezoelectric effects can be also ruled out, because we did not observe any measurable voltage generated between the Hall and four‐probe contacts of bent devices, which is not surprising given the fact that these effects require the crystal to be non‐centrosymmetric, or the applied strain to be inhomogeneous, neither of which is the case here.

### 2.4 Low‐Energy Raman Spectra as a Function of Strain

**Figure 3:**
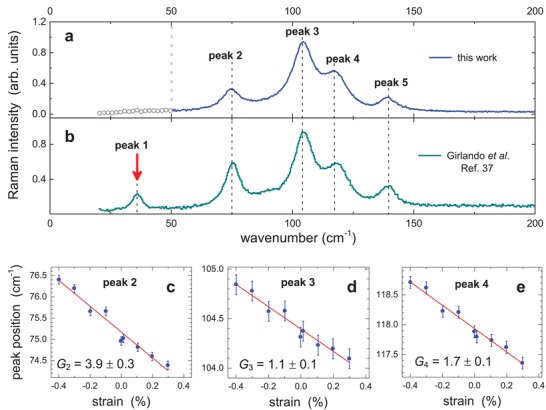
Low‐frequency region of Raman spectra of rubrene as a function of uniaxial strain. a) Raman spectrum of a thin rubrene crystal with the resolved peaks 2–5, marked by the thin dotted lines. Our spectrometer's resolution cutoff is at ≈50 cm^−1^, below which peaks cannot be resolved (indicated by the dotted gray line). b) Raman spectrum of a rubrene crystal reproduced for comparison from Girlando et al.^37^ The lowest‐energy mode at 35 cm^−1^ (peak 1) is indicated by the red arrow. c–e) The dependence of the position of Raman peaks 2 (75 cm^−1^), 3 (104.5 cm^−1^), and 4 (118 cm^−1^) on strain applied along the high‐mobility ***b***‐axis of rubrene. Compressive and tensile strains correspond to the negative and positive values, respectively. The solid red line is a linear fit, and the Grüneisen parameter, *G*, calculated from these fits via Equation (3) is indicated.

To better understand the relationship between the intrinsic mobility and strain in crystalline rubrene, we have also performed measurements of low‐frequency Raman spectra of thin flexible rubrene single crystals as a function of uniaxial strain (**Figure** **3**). Although selection rules make Raman scattering “blind” to molecular vibrations important for the charge transport, such as acoustic modes far from the center of the Brillouin zone (Γ‐point at *q* = 0), strain‐dependent Raman measurements can still serve as a proxy, assuming the modes far from the Γ‐point exhibit a similar behavior with strain.^35^ Specifically, the low‐energy region of Raman spectra (0–300 cm^−1^) is believed to be important for charge transport properties of organic semiconductors,^11^ as it contains low‐frequency intra‐ and intermolecular vibrations reflecting the off‐diagonal thermal disorder.^12^ It was recently proposed theoretically that the higher the frequency, *f*, of the lowest‐energy Raman peaks of an organic semiconductor, the better its charge carrier mobility.^36^ Alternatively, relatively low intensities (or a complete absence) of Raman peaks in this low‐frequency region would suggest that the material might be a high‐mobility organic semiconductor. If this proposal is correct, an increase (decrease) of *µ* of the crystal should be accompanied by an increase (decrease) of the frequency *f* of the low‐energy Raman modes that have the greatest electron–phonon coupling.

Figure 3a shows the low‐frequency region of the Raman spectrum of a thin rubrene crystal laminated on a semiflexible substrate (for technical details see the Experimental Section), with the main resolved peaks labeled “peaks 2–5”: 75, 104.5, 118, and 139.6 cm^−1^. The low‐frequency cutoff of our Raman spectrometer does not allow resolving features below ≈50 cm^−1^ (marked by the dotted gray line). Nevertheless, peaks 2–5 represent important molecular vibrations (for DFT simulations see Section S4 in the Supporting Information). For comparison, Figure 3b shows a low‐energy Raman spectrum of rubrene adopted from Girlando et al.,^37^ where measurements reveal the lowest‐energy peak at 35 cm^−1^. The dependence of the Raman peak positions on compressive and tensile strain applied along the ***b***‐axis of the crystal is shown in Figure 3c–e. All the resolved peaks show qualitatively similar behavior (for simplicity, only the data for peaks 2–4 are shown in the main text, with other peaks shown in Section S5 in the Supporting Information). It is clear from these measurements that low‐energy Raman peaks shift to higher (lower) frequencies with compressive (tensile) strain, which is indicative of the hardening (softening) of the corresponding phonon modes of the crystal.

To the best of our knowledge, this is the first measurement of Raman modes of an organic semiconductor as a function of uniaxial strain. In the past, Raman spectra of organic crystals were investigated under hydrostatic pressure in naphthalene,^38^, ^39^ anthracene,^38^, ^40^ tetracene,^41^ and pentacene^42^ (for a review, see ref. 11). It was found that mode frequencies increase with pressure, which is consistent with the strain‐dependent measurements performed here. These results suggest that interaction between the molecules strengthens, as they get closer with pressure. In the past, however, these pressure‐dependent measurements were not coupled with a concurrent characterization of the charge carrier mobility. In our work, strain‐dependent Raman measurements, *f*(ε), are correlated with the measurements of the charge carrier mobility as a function of strain, *µ*(ε), obtained in the same single crystals.

## 3 Discussion

The effect of strain on the frequency of Raman modes can be quantified by the so‐called Grüneisen parameter, *G*, which was originally defined as a fractional change of the mode's frequency, *δf*/*f*, per unit of the fractional change of the sample's volume, *δν*/ν, occurring under a hydrostatic pressure: $G\equiv \;-\frac{\nu }{f}\cdot \frac{\partial f}{\partial \nu }$.^43^ In measurements under uniaxial strain, the fractional change of the sample's volume is equivalent to the fractional change of its linear dimension, provided that the Poisson effect is not too strong. This allows us to redefine the Grüneisen parameter in terms of the applied strain ε (index *i* denotes a particular Raman mode)

(3) $$G_{i}\equiv \;-\frac{1}{f_{i}}\cdot \frac{\partial f_{i}}{\partial \varepsilon }$$

The Grüneisen parameter for peaks 2–4 is calculated from our data: *G*_2_ = 3.9 ± 0.3, *G*_3_ = 1.1 ± 0.1, and *G*_4_ = 1.7 ± 0.1.

Besides the consistent qualitative correlation between the effects of strain on mobility, *µ*(ε), and the low‐energy Raman modes, *f*(ε), our additional goal is to quantitatively analyze this correlation. In order to perform such an analysis, one needs to know the relationship between the charge carrier mobility and the frequency of the relevant Raman modes, *µ*( *f*  ). In the absence of precise theoretical relationship, below we resort to an approximation valid at high temperatures that treats molecular vibrations classically and uses the experimental temperature dependence of the charge carrier mobility in rubrene, *µ*(*T*), previously obtained via four‐probe FET and Hall effect measurements,^25^ and theoretically analyzed based on the model of off‐diagonal thermal disorder and transient carrier localization^9^, ^10^

(4) $$\mu (T)=\mu_{0}\cdot T^{\gamma },\;\gamma =-1.5$$

Here, *µ*_0_ is a constant, and exponent γ = –1.5 fits well the high‐temperature region (175–300 K) of *µ*(*T*) data relevant for the current study (Section S6, Supporting Information).

At high temperatures, energy of molecular vibrations can be treated classically, and thus the mechanical energy of a given vibrational mode can be expressed via thermal energy

(5) $$\frac{1}{2}M\omega^{2}\left(2\left\langle Q^{2}\right\rangle \right)≃\;k_{B}T$$

where *M* is the effective mass of the molecule in this vibrational mode, ω = 2*πf*(ε) is the strain‐dependent angular frequency of the mode, *Q* is the effective coordinate of the mode (with the mode's amplitude $Q_{amp}\equiv \sqrt{2〈Q^{2}〉}$), and *k*_B_ is the Boltzmann's constant. Equation (5) shows that the average amplitude of molecular vibrations depends both on temperature *T* and mode's frequency *f*

(6) $$Q_{\mathrm{amp}}=\;Q_{\mathrm{amp}}\left(T,\varepsilon \right)\;\propto \;\frac{\sqrt{T}}{f\left(\varepsilon \right)}$$

Since frequency is a function of strain, both strain ε and temperature *T* can be viewed as external stimuli directly controlling the intensity (the average amplitude) of off‐diagonal thermal disorder and thus indirectly controlling the charge carrier mobility, *µ* = *µ*(*T*, ε). In this sense, the effect of strain is equivalent to the effect of temperature: both influence thermal disorder according to Equation (6). For instance, a compressive strain increases the frequency of the low‐energy Raman modes, thus suppressing the amplitude *Q*_amp_ of molecular fluctuations and leading to an increase in the charge carrier mobility, similarly to the effect of decreasing temperature.

From here, in order to find the relationship between the charge carrier mobility and Raman mode frequency, *µ*( *f*  ), let us first find a variation in temperature, *δT*, that would correspond to the same variation of the amplitude of molecular vibrations, *δQ*_amp_, as that produced by a variation, *δε*, in strain. That is, we are looking for the condition, at which $\delta Q_{\mathrm{amp}}{|}_{\varepsilon }\equiv \;{\left(\frac{\partial Q_{\mathrm{amp}}}{\partial T}\right)}_{\varepsilon }\delta T$ is equal to $\delta Q_{\mathrm{amp}}{|}_{T}\equiv \;{\left(\frac{\partial Q_{\mathrm{amp}}}{\partial \varepsilon }\right)}_{T}\delta \varepsilon$. By taking partial derivatives of *Q*_amp_(*T*, ε) in Equation (6), we get

(7) $$\frac{\delta T}{T}\;=\;-\frac{2}{f}\;\frac{df}{d\varepsilon }\delta \varepsilon$$

Let us then express the variation in temperature, *δT*, in terms of *δμ* with the help of the experimental temperature dependence of carrier mobility (Equation (4)):

(8) $$\frac{\delta T}{T}\;=\;\frac{1}{\gamma }\;\frac{d\mu }{\mu }$$

Combining Equations (7) and (8), we obtain a relationship between the carrier mobility and the Raman mode frequency:

(9) $$\mu =const\cdot f^{-2\gamma },\;\;\;\mathrm{where}\;\gamma =-1.5$$

This equation suggests that the charge carrier mobility is a rather sensitive function of strain. Indeed, even if the frequency *f* of the relevant Raman mode is varied a little bit, the corresponding change in *µ* would be more significant, because *µ* ∝ *f* ^3^. For small variations in *f*, the corresponding variations in *µ* are roughly three times greater: $\frac{d\mu }{\mu }\;\approx 3\;\times \frac{df}{f}$. For instance, a strain factor of ≈70 obtained for *µ* in the charge transport measurements (Figure 2), indicating that a 70% increase of mobility occurs at 1% of compressive strain, implies that the corresponding relevant Raman mode should shift by about 23% $\left(\frac{df}{f}\;\approx \;0.23\right)$ under the same strain. This is of course much greater than the shifts observed in our Raman measurements for the resolved peaks (peaks 2–5 in Figure 3). However, we should keep in mind that one of the most important peaks in the low‐energy region of Raman spectrum of rubrene is the peak at 35 cm^−1^ (marked by the red arrow at Figure 3b),^37^ and it remained unresolved in our measurements because of the sensitivity cutoff of our Raman spectrometer at 50 cm^−1^. Given the proposal of the importance of lowest‐frequency vibrational modes for the charge carrier transport in organic semiconductors,^11^ we should estimate the Grüneisen parameter for this lowest‐energy Raman mode at 35 cm^−1^. This can be done by extrapolating the Grüneisen parameter measured for the resolved peaks. Such an extrapolation is possible via the well‐known scaling behavior of Grüneisen parameter, originally observed in pressure‐variable Raman measurements of molecular solids:^43^ *G* was found to roughly scale as 1/*f_i_* ^2^ with the Raman mode's frequency *f_i_*, suggesting that lower energy peaks should typically exhibit a greater relative shift with pressure. This scaling is a consequence of the coexistence of strong intramolecular and weak intermolecular forces characteristic of all molecular crystals. Based on this scaling law and *G_i_* we measured for the resolved peaks (*i* = 2, 3, and 4), we can approximately predict the Grüneisen parameter for the 35 cm^−1^ peak: $G_{35\;{\mathrm{cm}}^{-1}}\;=\;(3.9\;\pm \;0.3)\;\times \;\;{\left(\frac{75}{35}\right)}^{2}=\;18\;\pm \;1$, or $G_{35\;{\mathrm{cm}}^{-1}}\;=\;(1.1\;\pm \;0.1)\;\times \;\;{\left(\frac{104.5}{35}\right)}^{2}=\;10\;\pm \;1,$or $G_{35\;{\mathrm{cm}}^{-1}}\;=\;(1.7\;\pm \;0.1)\;\times \;\;{\left(\frac{118}{35}\right)}^{2}=\;19\;\pm \;1.$ These estimates are consistent with the 23% mode shift for the lowest energy peak expected from the above preliminary analysis. Of course, more rigorous work is needed on modeling the influence of strain on mobility through the vibrational frequencies of relevant Raman modes.

It must be noted that the mobility–strain relationship, *µ*(ε), observed in our experiment does not agree well with either of the two recent theoretical papers modeling the mobility–strain dependence in rubrene (Gali et al.,^44^ and Ruggiero et al.^45^). Furthermore, these theoretical works are not in a good agreement with each other. While Gali et al.^44^ do predict an increasing mobility for the charge transport along the high‐mobility axis of rubrene (here, the ***b***‐axis) with compressive strain applied either along or perpendicular to this axis, the predicted effect is smaller in magnitude compared to our experiment and almost completely lacks the anisotropy observed here. Indeed, Figures 5 and 7 of ref. 44 suggest that for the orthorhombic rubrene the ***b***‐axis mobility should increase by only about 8% per 1% of compressive strain, irrespective of the direction of strain application. On the contrary, for the same transport direction (along the high‐mobility ***b***‐axis), Ruggiero et al.^45^ predict that the effect of strain applied along the orthogonal direction (along the low‐mobility ***a***‐axis) should be much stronger than that of strain applied along the transport direction (Figure 7 of ref. 45). For the strain applied along the transport direction (the high‐mobility ***b***‐axis), Ruggiero et al.^45^ predict a very small effect: in pristine crystals, it has a negative sign (that is, *µ* slightly decreases with compressive strain), but becomes positive (while remaining very small in magnitude) with extrinsic disorder incorporated into the model. For any amount of extrinsic disorder, however, Ruggiero et al.^45^ predict a very small effect of strain applied along the high‐mobility ***b***‐axis, negligible compared to the effect of strain applied along the low‐mobility **a**‐axis. Given the molecular packing of rubrene, such an anisotropy is counterintuitive, it is not agreeing with the predictions of Gali et al.,^44^ and it is also at odds with the anisotropy of the strain effect observed in our experiment. These disagreements, both on qualitative and quantitative levels, clearly indicate that adequate theoretical description of the transport properties of organic semiconductors is still lacking, and further theoretical and experimental work in this direction is needed.

## 4 Conclusions

In conclusion, we have carried out Hall effect and low‐frequency Raman measurements in a crystalline organic semiconductor rubrene as a function of uniaxial strain. Our methodology allowed accessing for the first time the intrinsic dependences of the charge carrier mobility and low‐frequency molecular vibrations as a function of strain. Measurements were performed with strain applied along or perpendicular to the high‐mobility transport direction of rubrene (the direction of molecular stacks), revealing an anisotropic response of the FET and Hall mobilities to strain, consistent with the molecular packing of rubrene crystals. Both the (longitudinal) FET and Hall mobilities are found to be very sensitive to strain, with a significant strain factor *g* ≈ 70–100, for the strain along the high‐*µ* direction, indicating that *µ* can be enhanced by nearly a factor of two with a small compressive strain of 1%. Raman measurements revealed hardening (softening) of the low‐frequency molecular vibrations under compressive (tensile) strain, with the corresponding Raman modes at 75, 104.5, and 118 cm^−1^ shifting by about 1–4% per 1% of the applied strain, which is consistent with the observed changes in the charge carrier mobility with strain. To the best of our knowledge, this work is the first direct experimental demonstration of a correlation between the low‐frequency Raman modes and the charge carrier mobility of an organic semiconductor, obtained within the same system. Accurate magneto‐transport and Raman measurements, targeting the intrinsic response of charge transport and molecular vibrations to strain, are of crucial importance for future development of organic and flexible electronics. Such studies lay the foundation of strain engineering in high‐performance flexible organic electronics and can provide the necessary benchmark for theoretical studies, as well as help to introduce novel high‐performance devices in applications.

## 5 Experimental Section

*Device Fabrication*: Single crystals of rubrene with thicknesses in the range 100–300 nm were grown by physical vapor transport following published procudure.^22^ Selected crystals with well‐defined facets were laminated onto a PDMS‐coated PET substrates of dimensions 9 × 9 mm^2^. The thicknesses of the PDMS layer and the PET substrate are 1 and 40 µm, respectively. 5 nm thick Ti adhesion underlayer and 15 nm thick Au contacts were sputtered through a contact shadow mask on PDMS prior to the crystal lamination. The devices are loaded in a custom‐designed miniature strain stage that can be used to apply a calibrated uniaxial mechanical strain of up to ±5% (Figure 1e). Device wiring is done using a gold wire of 25 µm in diameter and a conducting silver paint.

*Electric Measurement*: All electric measurements were carried out at room temperature in a coarse dry vacuum (10^−2^ Torr). Keithley K6221 current source, Hewlett‐Packard HP34401A multimeter, and Keithley K6514 electrometer were used to set the excitation source–drain current, to measure the source–drain voltage, and to measure the four‐probe voltage, respectively. In *ac*‐Hall measurements, in‐phase and out‐of‐phase components of Hall voltage, *V*_Hall_^ip^ and *V*_Hall_^op^, were registered by Stanford Research SR830 lock‐in amplifier tuned into the frequency of an *ac* magnetic field applied perpendicular to the FET's channel carrying a *dc* source–drain current. The net Hall voltage, *V*_Hall_, is determined as the absolute value of the vector with these components: $V_{\mathrm{Hall}}\equiv \sqrt{{\left(V_{\mathrm{Hall}}^{\mathrm{ip}}\right)}^{2}+{\left(V_{\mathrm{Hall}}^{\mathrm{op}}\right)}^{2}}$, following the previously developed procedure.^33^ The *ac*‐*B* field of frequency 0.5–1.5 Hz and an r.m.s. magnitude of 0.23 T was generated by rotating an assembly of permanent Nd magnets. Finite width of the voltage probes in the four‐probe contact geometry may lead to a longitudinal channel shunting and related inaccuracies in the four‐probe conductivity and mobility calculations when the conventional equations are used, as investigated in detail by Choi et al.^46^ For the structures used in this work, these corrections are minor. Nevertheless, they were taken into account by the procedure developed in ref. 46. Application of callibrated uniaxial strain was achieved by using a custom‐designed strain stage that holds a semirigid substrate of unstrained length, *l*_0_, between its stationary and sliding jaws and bends it with a vise‐like motion, producing a displacement, Δ*l*, of the sliding jaw controlled by a micrometric screw. The length *l*_0_ of a flat (unbent) substrate and the edge‐to‐edge distance (along the chord) of a bent substrate, *l*_0_ − Δ*l*, are used to calculate the bending radius, *R*, near the center of the substrate (Section S2, Supporting Information).^34^ The strain in % is then calculated as ε = $\frac{t}{2R}$ × 100%, where *t* = 41 µm is the net thickness of the composite substrate (PET coated with PDMS).

*Raman Measurements*: Flexible 125 µm thick PEN substrates were used for crystal support. To block off the Raman signal from PEN, the substrates were coated with a 50 nm thick Au film by thermal evaporation. To improve the adhesion of rubrene crystals, an ≈10 nm thick Cytop was spin‐coated over gold. Raman measurements were performed with HORIBA LabRAM HR Evolution spectrometer using an incident laser of wavelength 632.8 nm, which provides slightly sub bandgap excitation of rubrene, necessary to achieve a good trade‐off between the fluorescence of rubrene and Raman signal. The incident laser power was kept below 8 mW to avoid sample's heating. During measurements, the laser spot was continuously rastered over the area of 10 × 10 µm^2^ to distribute the incident power and collect an averaged signal. The error in the Raman peak position measurements was ≈0.1 cm^−1^, and the error in the determination of Grüneisen parameter was mostly due to the standard deviation of the slope of the linear fits in Figure 3c–e.

## Conflict of Interest

The authors declare no conflict of interest.

## Supporting information

Supporting Information
