# Supplementary material for: A Large Anisotropic Enhancement of the Charge Carrier Mobility of Flexible Organic Transistors with Strain: A Hall Effect and Raman Study
Source: Adv Sci (Weinh). 2019 Nov 13;7(1):1901824. doi: 10.1002/advs.201901824 (PMC6947506; doi:10.1002/advs.201901824)
Supplement: Supplementary file 1 — Supporting Information [file ADVS-7-1901824-s001.pdf]

Copyright WILEY-VCH Verlag GmbH & Co. KGaA, 69469 Weinheim, Germany, 2019.

Supporting Information

for *Adv. Sci.,* DOI: 10.1002/advs.201901824

A Large Anisotropic Enhancement of the Charge Carrier Mobility of Flexible Organic Transistors with Strain: A Hall Effect and Raman Study

*Hyun Ho Choi, Hee Taek Yi, Junto Tsurumi, Jae Joon Kim, Alejandro L. Briseno, Shun Watanabe, Jun Takeya, Kilwon Cho, and Vitaly Podzorov**

Supplementary Information

A Large Anisotropic Enhancement of the Charge Carrier Mobility of Flexible Organic Transistors with Strain: a Hall Effect and Raman Study

Oct. 22, 2019

Hyun Ho Choi [1,2], *Hee Taek Yi* [1], *Junto Tsurumi* [3,4,5], *Jaejoon Kim* [6], *Alejandro L. Briseno* [6,†],

Shun Watanabe [3,4,5], *Jun Takeya* [3,4,5], *Kilwon Cho* [7], and *Vitaly Podzorov* [1,*]

[1] Department of Physics, Rutgers University, Piscataway, New Jersey 08854, USA.

[2] School of Materials Science and Engineering & Engineering Research Institute, Gyeongsang National University, Jinju 52828, Korea.

[3] International Center of Materials Nanoarchitectonics, National Institute for Materials Science (NIMS), 1-1 Namiki, Tsukuba 305-0044, Japan.

[4] Material Innovation Research Center (MIRC) and Department of Advanced Materials Science, Graduate School of Frontier Sciences, The University of Tokyo, 5-1-5 Kashiwanoha, Kashiwa, Chiba 277-8561, Japan.

[5] National Institute of Advanced Industrial Science and Technology (AIST), The University of Tokyo Advanced Operando-Measurement Technology Open Innovation Laboratory (OPERANDO-OIL), AIST, 5-1-5 Kashiwanoha, Kashiwa, Chiba 277-8561, Japan.

[6] Department of Polymer Science and Engineering, University of Massachusetts Amherst, Amherst, Massachusetts 01003, USA.

[7] Center for Advanced Soft Electronics and Department of Chemical Engineering, Pohang University of Science and Technology (POSTECH), Pohang 37673, Republic of Korea.

[*] Corresponding author's e-mail address: podzorov@physics.rutgers.edu

[†] Current address: Department of Chemistry, The Pennsylvania State University, University Park, PA 16803, USA.

1. The crystallographic orientation of rubrene single crystals with respect to the contacts.

Rubrene single crystals have anisotropic optical properties, with the absorption coefficient reaching its maximum (minimum) for the incident light polarized along the high-mobility ***b***-axis (low-mobility ***a***-axis) of the crystal.[1] We use this property to determine the crystallographic orientation of the crystals in our assembled rubrene devices on transparent substrates (for device structure see **Fig. 1** of the main text) by shining a linearly polarized light through them and recording the intensity of the transmitted light. A monochromatic light (λ = 460 nm, well absorbed by rubrene) at a normal incidence to the crystal's (***a***,***b***)-facet was used in these measurements.

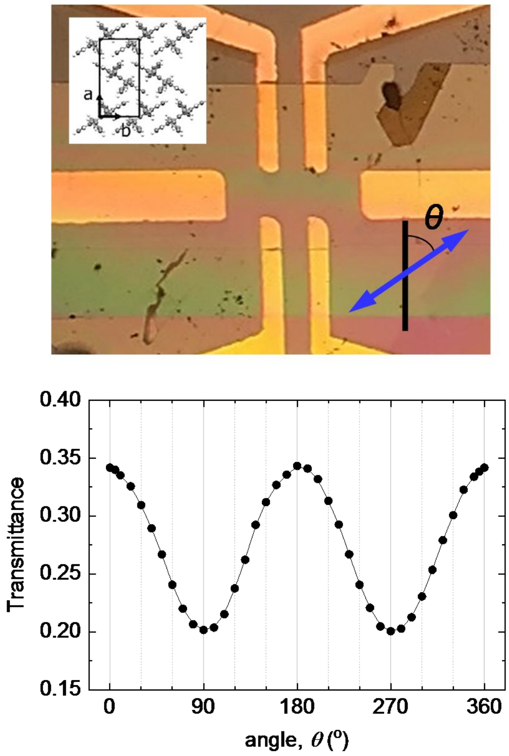

Figure S1. Determining the crystal orientation via transmission of polarized light. Optical transmittance of a linearly polarized light through a thin rubrene single crystal laminated on a transparent substrate as a function of polarization angle, θ, measured with respect to the W-edge of the source and drain contacts (the vertical direction in the photograph). The transmittance maxima and minima are observed at the angle $\theta = n\pi$ and $(n+1/2)\pi$, respectively, where n = 0, 1, 2, …, which correspond to the low-mobility ***a***-axis and the high-mobility ***b***-axis of the crystal, respectively.[1]

2. Calculating strain exerted on the crystal by bending the supporting substrate.

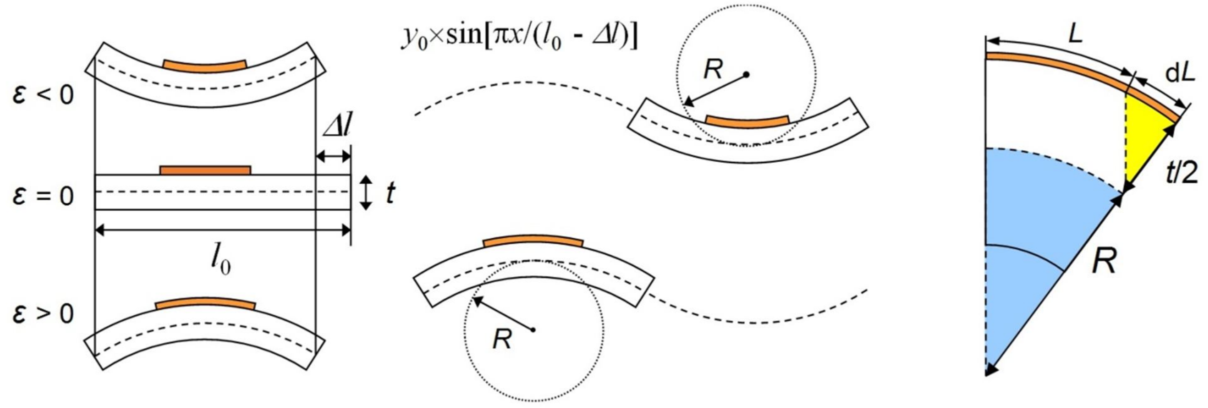

Figure S2. **Geometrical parameters used in the calculation of strain in bent devices.** (**Left**) Schematics of a substrate of thickness *t* with a layer of material deposited on its top surface (brown). The original (unstrained) length of the substrate is l_0. The substrate can be bent to an edge-to-edge length l_0 - Δl (measured along the straight line connecting the edges). (**Middle**) The shape of a bent substrate is usually close to a sinusoidal function, $y(x) = y_0 \cdot \sin[\pi x/(l_0 - \Delta l)]$, where *x* and *y* are the coordinates in the plane of the drawing, the amplitude $y_0 = y_0(l_0, \Delta l)$ is itself a function of l_0 and Δl, and the wavelength of the sine function is $2(l_0 - \Delta l)$ (see, e.g., Ref. [2] for more details). (**Right**) A segment of the substrate of thickness *t*, with a device on top (brown), bent to a radius *R*.

Figure S2 shows a substrate with a device, bent to an edge-to-edge distance l_0 - Δl (this is the distance between the edges of the substrate measured along the straight line connecting them). In our strain stage, we vary the horizontal displacement Δl in increments of approximately 60 - 65 µm by adjusting the micrometric screw, while the original length of the substrate l_0 remains the same (**Fig. 1 e, f** of the main text). In this process, the edges of the substrate are not clamped. Thus, when the substrate is forced to bend with a vise-like motion of the jaws of the stage, the edges are free to rotate. As described in detail by Park *et al.*[2], the shape of the substrates bent in such a way is most closely described by a sinusoidal function, $y = y_0 \cdot \sin[\pi x/(l_0 - \Delta l)]$, where *x* and *y* are the two-dimensional coordinates in the plane of the drawing (**Fig. S2**), and the amplitude $y_0 = y_0(l_0, \Delta l)$ is itself a function of l_0 and Δl. This amplitude y_0 can be determined by requiring that the length of the sine curve between $x = 0$ and $x = l_0 - \Delta l$ is maintained at l_0:

$$\int_{left\ edge}^{right\ edge} \sqrt{(dx)^2 + (y'_x dx)^2} = l_0 \qquad \text{S1}$$

Here, the integral is a path integral taken along the sine curve between the edges of the substrate (along the dotted line in the middle panel of **Fig. S2**). Expressed in terms of l_0, Δl and y_0, this Eq. becomes:

$$\frac{l_0 - \Delta l}{\pi} \cdot \int_0^{\pi} \sqrt{1 + \left(\frac{\pi y_0}{l_0 - \Delta l}\right)^2 \cdot cos^2(z)}\, dz \ = \ l_0 \qquad \text{S2}$$

To determine $y_0 = y_0(l_0, \Delta l)$, which we will need in further calculations, the integral can be either computed numerically or approximated by replacing the square root with its Taylor expansion to the first couple of terms. Such an approximation is valid for small bending ($\Delta l << l_0$ and $y_0 << l_0$), when the second term under the square root is much smaller than unity. After that, the integral can be taken analytically, leading to the following expression for y_0:

$$y_0 \ \approx \ \frac{2}{\pi} \cdot l_0 \cdot \left(\frac{\Delta l}{l_0}\right)^{\frac{1}{2}}, \quad for\, \Delta l \ \ll \ l_0 \ and \ y_0 \ll \ l_0. \qquad \text{S3}$$

The experimentally determined l_0 and Δl, as well as the calculated y_0 can now be used to calculate the bending radius R at the center of the substrate. R is given by the reciprocal curvature of the substrate. Generally, the curvature, κ, of a smooth continuous plane function $y(x)$ can be found as:

$$\kappa(x) \ = \ \frac{|y''|}{(1 + (y')^2)^{3/2}} \qquad \text{S4}$$

For a sine curve, this leads to the following expression for bending radius R in the middle of the substrate (at the point of maximum $y(x)$, $x_{max} = (l_0 - \Delta l)/2$):

$$R \ \equiv \ \frac{1}{\kappa} \ = \ \frac{(l_0 - \Delta l)^2}{\pi^2 y_0} \ \approx \ \frac{1}{2\pi} \cdot \frac{(l_0 - \Delta l)^2}{\sqrt{l_0 \cdot \Delta l}} \qquad \text{S5}$$

The corresponding strain (in %) exerted on the material deposited at the surface of the substrate (shown in brown in **Fig. S2**) is:

$$\varepsilon = \frac{t}{2R} \cdot 100\% \qquad \text{S6}$$

3. *ac*-Hall effect measurements of flexible rubrene OFETs at different strains (raw data).

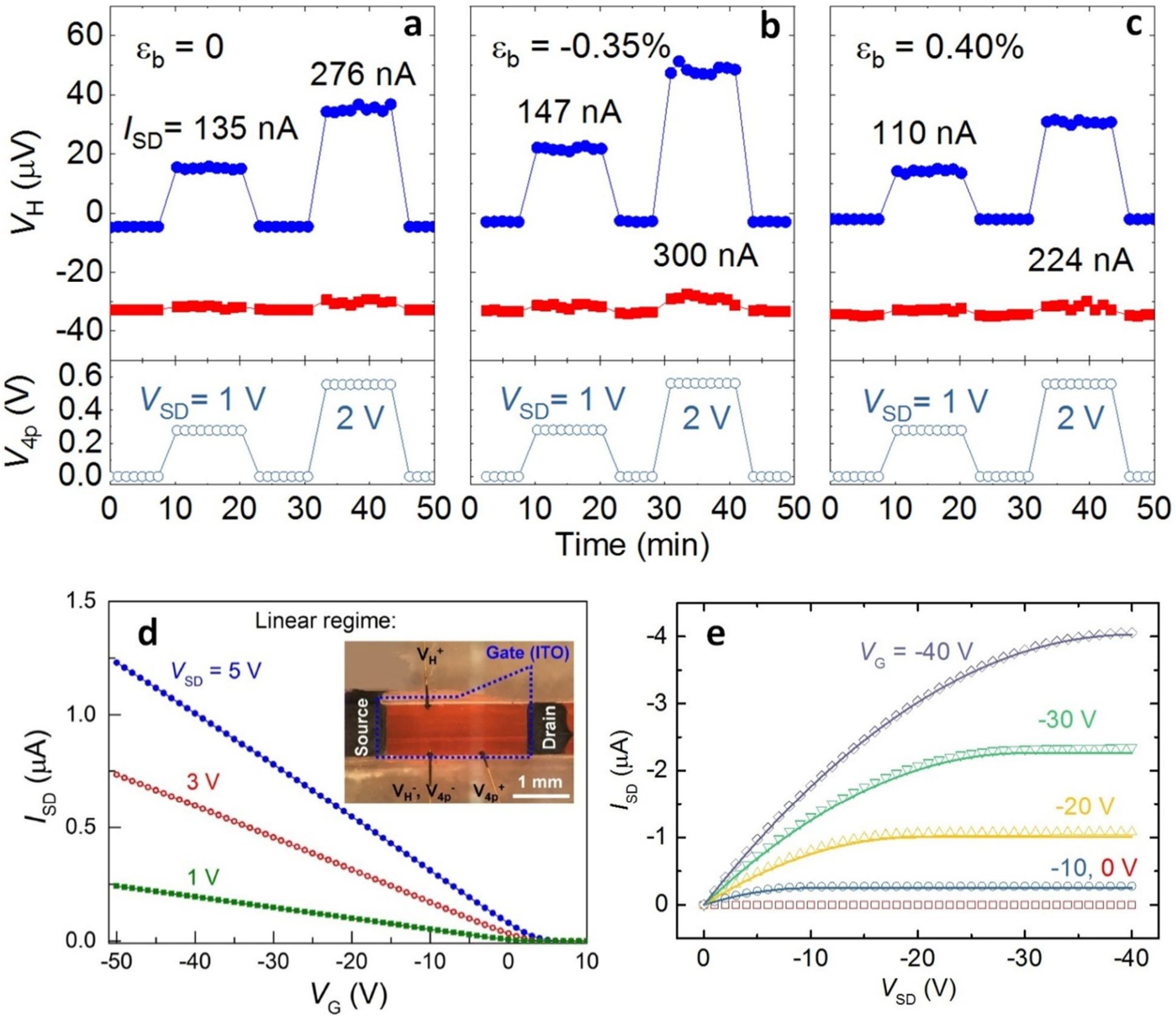

Figure S3. Measurements of *ac*-Hall and four-probe voltages in a bendable single-crystal rubrene OFET (panels a, b, c). The in-phase (blue solid circles), out-of-phase (red solid squares) components of the Hall voltage V_H, as well as the four-probe voltage V_{4p} (open circles) are shown. The *dc* source-drain current I_{SD} (indicated in the upper portions of panels **a**, **b** and **c**) flows along the high-mobility ***b***-axis of rubrene, with strain applied along the same direction. The *on* and *off* switching cycles correspond to I_{SD} being intentionally turned *on* and *off* (not necessarily periodically) to establish a zero-current baseline of the Hall voltage (that is, the voltage presented between the Hall probes at $I_{SD} = 0$). Here, only the data for strain magnitudes ε = 0, -0.35% and 0.4% are shown as examples. The gate voltage in this measurement was V_G = - 40 V; the source-drain voltage V_{SD} was either 1 or 2 V (as indicated in the lower portions of panels **a**, **b** and **c**). The corresponding calculated Hall and four-probe longitudinal FET mobilities, μ_{Hall}

and μ_{FET}, are shown in **Fig. 2** of the main text. **Typical transfer and output characteristics of single-crystal rubrene FETs (panels d, e).** Device parameters: L = 2.5 mm, L/W = 2.76, C_i = 1.24 nF·cm^{-2}. The symbols are the experimental data; the solid lines in panel **e** are fits with the Shockley FET equations with the mobility μ = 11.2 cm^{2}V^{-1}s^{-1} (for details, see Refs. [4] and [14] of the main text).

Figure S3 above gives a few examples of the raw *ac*-Hall effect data (that is, the in-phase and out-of-phase Hall voltage components, as well as the longitudinal four-probe voltage) recorded under different uniaxial strains applied along the high-mobility ***b***-axis of the rubrene crystal (panels **a**, **b** and **c**). These data are obtained in a thin, flexible single-crystal rubrene OFET (see the main text). In these measurements, an *ac* magnetic field of a low frequency (0.5 - 1.5 Hz) and an r.m.s. magnitude of B_{rms} = 0.23 T was used, with a lock-in detection of the corresponding Hall voltage. The negative (positive) values of strain ε correspond to the compressive (tensile) strain.

Figure S3 also shows an example of typical linear-regime transfer $I_{SD}(V_G)$ (panel **d**) and output $I_{SD}(V_{SD})$ (panel **e**) characteristics of single-crystal rubrene FETs. These characteristics belong to a rigid FET based on a bulky crystal (as shown in the inset in panel **d**), although the bendable ultra-thin rubrene FETs exhibit similar behavior.

4. Assignment of low-frequency Raman modes to molecular vibrations of rubrene.

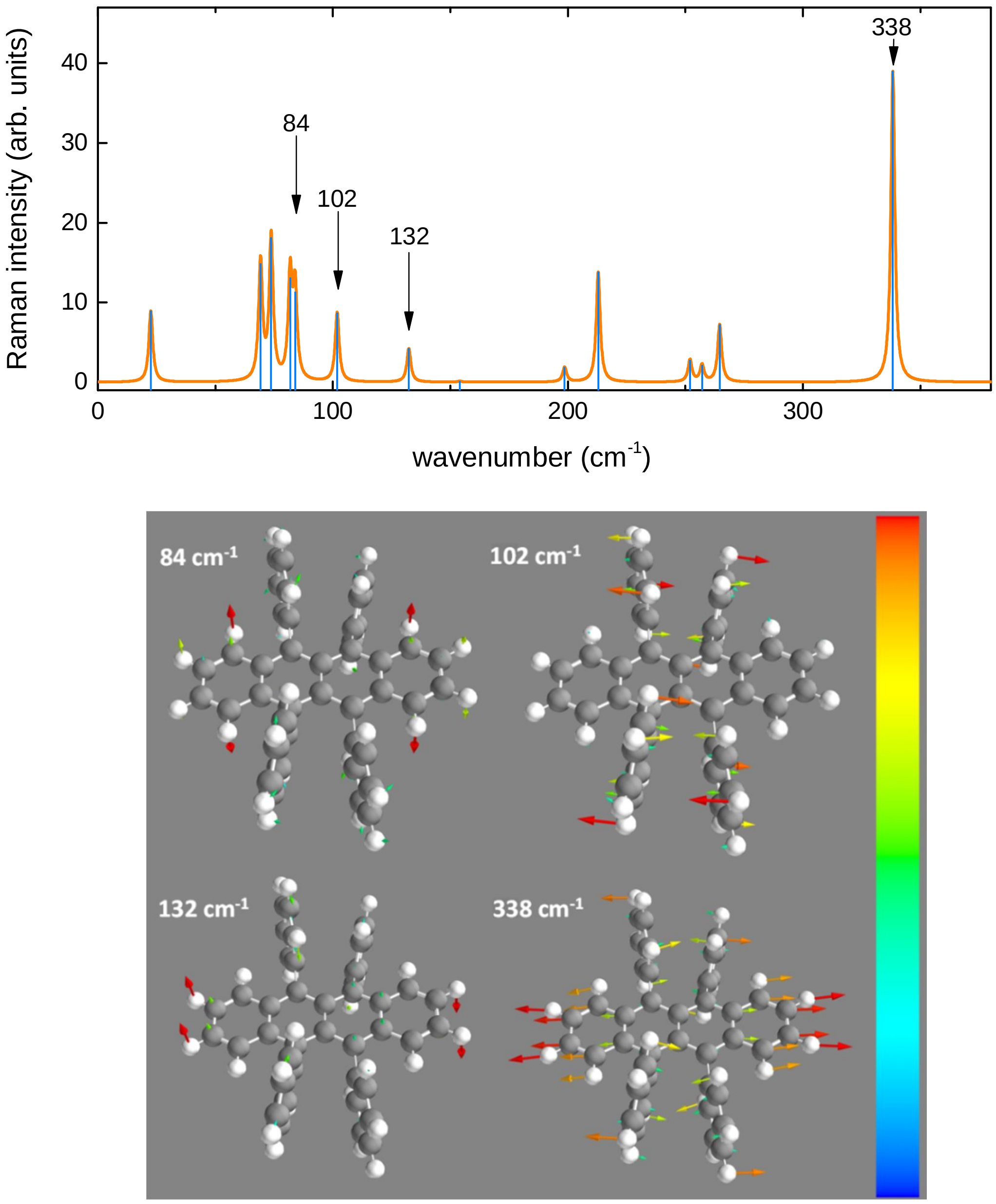

Figure S4. A simulated Raman spectrum of crystalline rubrene with a few important vibrational modes. (**Top**) The simulated low-frequency Raman spectrum of rubrene. (**Bottom**) A few examples of vibrational modes corresponding to the peaks indicated in the top panel with black arrows. The atomic motion is shown by the colored arrows (color and length of the arrows indicate the amplitude of the motion). The simulations were carried out using Gaussian 09 package with DFT at the B3LYP/6-311G(d,p) level.

5. Low-frequency Raman modes of rubrene as a function of uniaxial strain.

The low-frequency region of the Raman spectra of thin rubrene single crystals is shown in the main text (**Fig. 3**). The four peaks (labeled "peak 2 - 5") that we could resolve with our Raman spectrometer are at 75, 104.5, 118 and 139.6 cm^{-1}. **Figure S5** below shows how these peaks shift with strain. A tendency for the peak's frequency to increase under compressive and decrease under tensile strain was observed for all these Raman modes.

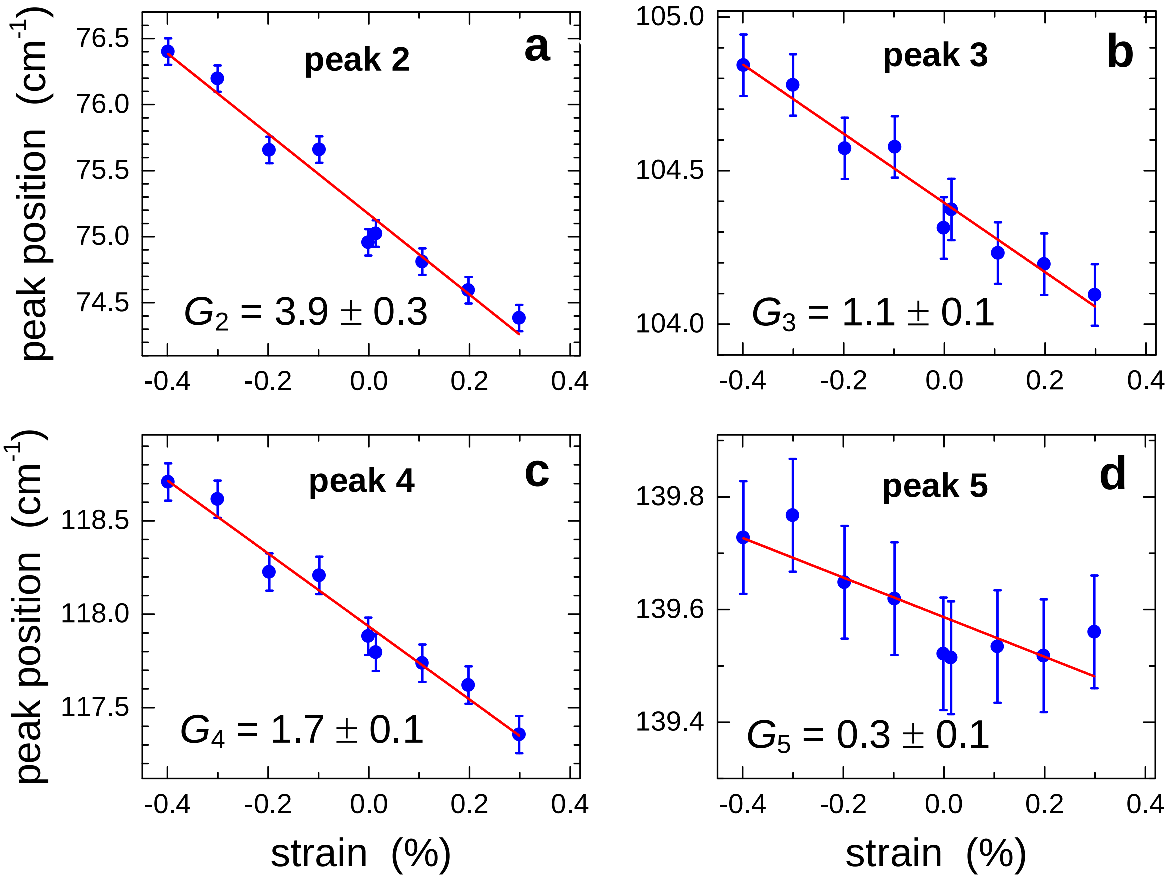

Figure S5. The low-energy Raman peak positions as a function of strain in rubrene. The Raman peaks 2 - 5 (see **Fig. 3** of the main text) are shown (panels **a-d**, respectively). Strain is applied along the high-mobility ***b***-axis of a rubrene single crystal (negative and positive values correspond to compressive and tensile strain, respectively). The error in the Raman peak position measurements was ~ 0.1 cm^{-1}, and the error in the determination of Grüneisen parameter was mostly due to the standard deviation of the slope of the linear fit (solid red line). The Grüneisen parameters *G* (indicated) is calculated from these fits for each Raman mode. This parameter, defined by Eq. 3 of the main text, is a relative shift of the peak's frequency in % per 1 % of applied strain.

6. Fitting the experimental temperature dependence of mobility in rubrene.

The experimental temperature dependence of the charge carrier mobility, $\mu(T)$, in rubrene was previously studied in single-crystal OFETs via four-probe FET and Hall effect measurements,[3] and it was then theoretically analyzed based on the model of off-diagonal thermal disorder and transient carrier localization, which led to the following expression:[4,5,6]

$$\mu(T) = \mu_0 \cdot T^{\gamma}, \quad \gamma = -1.5 \qquad \text{S7}$$

Here, μ_0 is a constant, and exponent $\gamma = -1.5$ fits the high-temperature region of $\mu(T)$ data at 175 - 300 K very well. Indeed, as can be seen in **Fig. S6**, the orange curve that corresponds to the power exponent $\gamma = -1.5$ describes the data much better than $\gamma = -1$ or -2 (green and blue curves). This range of temperatures is particularly relevant to our current study under a varied strain that has been carried out at room temperature.

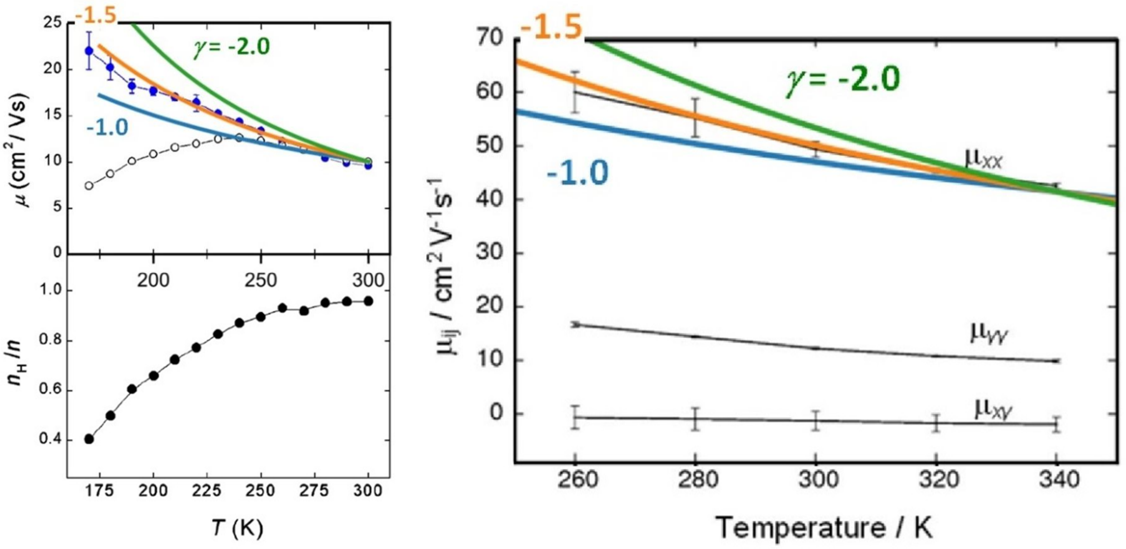

Figure S6. Temperature dependence of the hole mobility in rubrene. (Left) Experimental temperature dependence of the hole mobility in rubrene OFETs (blue solid circles, adopted from Ref. [3]). **(Right)** Theoretically calculated hole mobility in rubrene (black solid lines, adopted from Ref. [6]). The colored solid lines are the fits with a power-law dependence $\mu = \mu_0 \cdot T^{\gamma}$, with the exponents $\gamma = -2$ (green), -1.5 (orange) and -1.0 (blue). The best fit is delivered by the power exponent $\gamma = -1.5$.

References:

1. Irkhin, P., Ryasnyanskiy, A., Koehler, M., Biaggio, I. Absorption and photoluminescence spectroscopy of rubrene single crystals. *Phys. Rev. B* **86**, 085143 (2012).

2. Park, S.-I., Ahn, J.-H., Feng, X., Wang, S., Huang, Y., Rogers, J. A. Theoretical and Experimental Studies of Bending of Inorganic Electronic Materials on Plastic Substrates. *Adv. Funct. Mater.* **18**, 2673-2684 (2008).

3. Podzorov, V., Menard, E., Rogers, J. A., Gershenson, M. E. Hall effect in the accumulation layers on the surface of organic semiconductors. *Phys. Rev. Lett.* **95**, 226601 (2005).

4. Troisi, A., Orlandi, G. Charge-Transport Regime of Crystalline Organic Semiconductors: Diffusion Limited by Thermal Off-Diagonal Electronic Disorder. *Phys. Rev. Lett.* **96**, 086601 (2006).

5. Fratini, S., Mayou, D., Ciuchi, S. The Transient Localization Scenario for Charge Transport in Crystalline Organic Materials. *Adv. Funct. Mater.* **26**, 2292-2315 (2016).

6. Troisi, A. Dynamic disorder in molecular semiconductors: Charge transport in two dimensions. *J. Chem. Phys.* **134**, 034702 (2011).
